# Correction: Regulatory roles of *Escherichia coli* 5' UTR and ORF-internal RNAs detected by 3' end mapping

**DOI:** 10.7554/eLife.69260

**Published:** 2021-04-13

**Authors:** Gisela Storz, Philip P Adams, Gabriele Baniulyte, Caroline Esnault, Kavya Chegireddy, Navjot Singh, Molly Monge, Ryan K Dale, Joseph T Wade

Adams PP, Baniulyte G, Esnault C, Chegireddy K, Singh N, Monge M, Dale RK, Storz G, Wade JT. 2021. Regulatory roles of *Escherichia coli* 5' UTR and ORF- internal RNAs detected by 3' end mapping. *eLife* **10**:e62438. doi: 10.7554/eLife.62438.

Published 18, January 2021

In the original version of this manuscript, we used a previously published sequence of a ChiX northern oligonucleotide probe (Moon and Gottesman, 2011; PMID:22040174, doi.org/10.1111/j.1365-2958.2011.07907.x). We have discovered this sequence was mislabeled in that study and is actually complementary to the SdsR sRNA. Therefore, we have re-probed the northern blot in Figure 6B with a probe of the correct sequence. This blot now shows ChiX levels decrease with the overexpression of ChiZ. This does not change the main conclusion of the figure, which provides evidence for the ChiZ sRNA sponging of ChiX and the consequences of this for the ChiX target *chiP*.

Figure 6 has been updated, and the sequence of the ChiX oligonucleotide has been corrected in Supplementary file 4. Additionally, the text has been changed by a few words in three places to correct statements that are no longer accurate.

The following two sentences have been corrected in the text (changes are underlined):

“Upon ChiZ overexpression in the WT background, we observed increased levels of the chiP mRNA, without a change in ChiX levels (Figure 6B).”

to

“Upon ChiZ overexpression in the WT background, we observed increased levels of the *chiP* mRNA, with a reciprocal change in ChiX levels (Figure 6B).”

“In this model, when chitooligosaccharides need to be imported, ChiZ prevents ChiX from base pairing, but does not promote degradation of this sRNA. When metabolic needs shift, ChiX could be released from ChiZ allowing ChiX to regulate *chiP* and other targets. This may work in competition, conjunction, or at separate times from the *chbBC* intergenic mRNA sequence, which also sponges ChiX, but results in ChiX decay (Overgaard et al., 2009).”

to

“In this model, when chitooligosaccharides need to be imported, ChiZ prevents ChiX from base pairing, and promotes degradation of ChiX. When metabolic needs shift, the levels of ChiZ could decrease, allowing ChiX to regulate *chiP* and other targets. This may work in competition, conjunction, or at separate times from the *chbBC* intergenic mRNA sequence, which also sponges and promotes decay of ChiX (Overgaard et al., 2009).”

The Figure 5–figure supplement 1 legend has been corrected (changes are underlined):

“Secondary structures of ChiX (A) and IspZ (B) predicted by sfold”

to

“Secondary structures of ChiZ (A) and IspZ (B) predicted by sfold”

The corrected Figure 6 is shown here:

**Figure 1:**
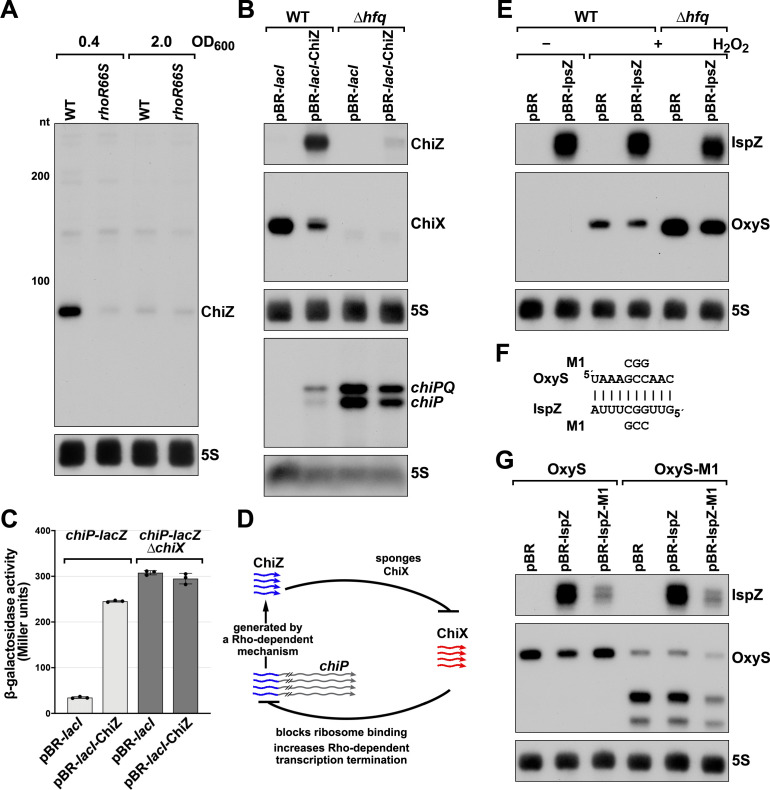


The originally published Figure 6 is also shown here for reference:

**Figure 2:**
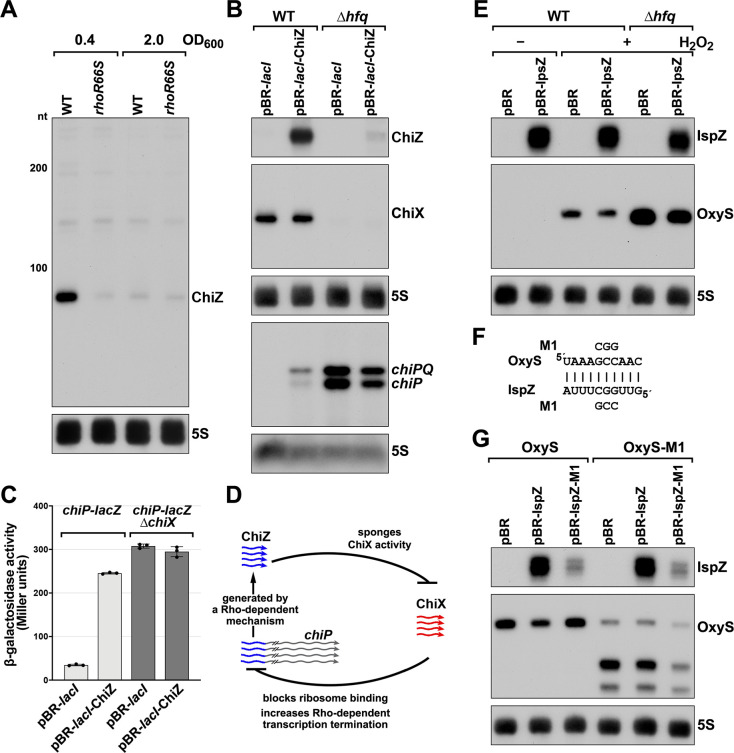


The article has been corrected accordingly.

